# Tunable Electronic Properties of Nitrogen and Sulfur Doped Graphene: Density Functional Theory Approach

**DOI:** 10.3390/nano9020268

**Published:** 2019-02-15

**Authors:** Ji Hye Lee, Sung Hyun Kwon, Soonchul Kwon, Min Cho, Kwang Ho Kim, Tae Hee Han, Seung Geol Lee

**Affiliations:** 1 Department of Organic Material Science and Engineering, Pusan National University, 2, Busandaehak-ro 63beon gil, Geumjeong-gu, Busan 46241, Korea; iciti5425@pusan.ac.kr (J.H.L.); rnjstjdgus5@hanmail.net (S.H.K.); 2 Department of Civil and Environmental Engineering, Pusan National University, 2, Busandaehak-ro 63beon gil, Geumjeong-gu, Busan 46241, Korea; sckwon@pusan.ac.kr; 3 Division of Biotechnology, Advanced institute of Environment and Bioscience, College of Environmental and Bioresource Sciences, Chonbuk National University, Iksan 54596, Korea; cho317@jbnu.ac.kr; 4 School of Materials Science and Engineering, Pusan National University, 2, Busandaehak-ro 63 Beon-gil, Geumjeong-gu, Busan 46241, Korea; khkim@pusan.ac.kr; 5 Department of Organic and Nano Engineering, Hanyang University, Seoul 04763, Korea

**Keywords:** co-doping, graphene, electronic structure, density functional theory, tunable electronics

## Abstract

We calculated the band structures of a variety of N- and S-doped graphenes in order to understand the effects of the N and S dopants on the graphene electronic structure using density functional theory (DFT). Band-structure analysis revealed energy band upshifting above the Fermi level compared to pristine graphene following doping with three nitrogen atoms around a mono-vacancy defect, which corresponds to p-type nature. On the other hand, the energy bands were increasingly shifted downward below the Fermi level with increasing numbers of S atoms in N/S-co-doped graphene, which results in n-type behavior. Hence, modulating the structure of graphene through N- and S-doping schemes results in the switching of “p-type” to “n-type” behavior with increasing S concentration. Mulliken population analysis indicates that the N atom doped near a mono-vacancy is negatively charged due to its higher electronegativity compared to C, whereas the S atom doped near a mono-vacancy is positively charged due to its similar electronegativity to C and its additional valence electrons. As a result, doping with N and S significantly influences the unique electronic properties of graphene. Due to their tunable band-structure properties, the resulting N- and S-doped graphenes can be used in energy and electronic-device applications. In conclusion, we expect that doping with N and S will lead to new pathways for tailoring and enhancing the electronic properties of graphene at the atomic level.

## 1. Introduction

Graphene consists of two-dimensional sheets of sp^2^-bonded carbon atoms arranged in a honeycomb lattice [1,2,3]. It is a zero bandgap semiconductor or semimetal with a large surface area of 2630 m^2^ g^−1^ [4,5], which is larger than other carbon-based materials [5,6]. Graphene also has exceptional charge-carrier mobility of 2 × 10^5^ cm^2^ V s^−1^ [7], good thermal conductivity of ~5000 W m^−1^K^−1^ [8] and high mechanical strength with ~1 TPa of Young’s modulus [9]. Due to its fascinating properties, graphene is considered to be a promising candidate material for applications in a wide range of fields, such as nanoelectronics [10], optoelectronics [11], energy-storage and conversion devices [12,13,14], sensors [15], and catalysts [16]. It is essential that the intrinsic electronic properties of graphene are tailorable for use in a range of nanoelectronics devices. Tremendous effort has been dedicated to the tuning of the electronic properties of graphene, and various techniques have been proposed [17,18,19,20,21,22,23,24,25,26,27,28,29,30,31,32,33]. As the zero bandgap, at the Fermi level, is attributed to the sub-lattice symmetry of the graphene structure, breaking this symmetry will induce bandgap widening. Substitutional doping and the formation of atomistic defects such as vacancies are simple and effective methods for opening the bandgap and altering the band structure of graphene. The band structure can subsequently be tuned by controlling the degree of heteroatom doping or the number of vacancies. The electronic properties of graphene have been found to change considerably when doped with single heteroatoms, such as B, N, O, P, or S [17,18,19,23,24,28,29,30,33]. Because of the relative differences in the electronegativities of the atomic dopants with respect to that of C, heteroatom doping is expected to induce changes in the band structure, charge distribution, and magnetic properties of graphene. Both experimental and theoretical studies have revealed that graphitic N atoms lead to n-type behavior, whereas pyridinic and pyrrolic N atoms give rise to p-type behavior [22,23,24]. Therefore, controlling the bonding configurations of the N atoms in graphene may provide a mechanism for tuning the electronic characteristics from n-type to p-type. Recently, co-doping with multiple heteroatoms has become popular because co-doping creates a unique, synergistically coupled, electronic structure. However, there are few reports that provide a fundamental understanding of the alternating electronic structure and accompanying performance of co-doped graphene [25,26,27,28,31,32,33]. Among the atoms possible as N co-dopants, the S atom is considered to be an attractive doping material due to its similar electronegativity and van der Waals radius to those of C, while possessing two lone pairs of electrons. Herein, we present a spin-polarized density functional theory (DFT) study on the electronic properties of N- and S-doped graphene in which we characterize changes in band structure and charge-density distribution by controlling the concentrations of the N and S dopants.

## 2. Computational Details

First-principles density functional theory (DFT) calculations were carried out using the Vienna Ab Initio Simulation Package (VASP) [34,35]. Geometries were optimized, and the total energies and forces were calculated using a planewave basis set with the projector augmented wave (PAW) method [36]. The generalized gradient approximation (GGA) with the Perdew–Burke–Ernzerhof (PBE) exchange-correlation functional [37] was used, and the planewave cutoff energy was set to 500 eV; the GGA-PBE functional has been successfully used to describe carbon-based systems [38,39,40,41,42,43]. All structures were optimized such that the total energy converged to less than 1.0 × 10^−6^ eV per atom and the maximum force converged to below 0.05 eV Å^−1^. The graphene model used in our simulation consisted of a 12.3 × 12.3 × 15.0 Å, 5 × 5 supercell with a vacuum thickness of 15 Å, which avoids interference between adjacent graphene layers. Brillouin-zone integrations were carried out using a 4 × 4 × 1 Monkhorst–Pack Κ-point grid. The effects of van der Waals (vdW) interactions were included using the empirical DFT-D3 correction within the Grimme scheme [44]. All atomic charge distributions in our study were calculated by Mulliken population analysis from Materials Studio [45,46].

## 3. Results and Discussion

**Figure 1:**
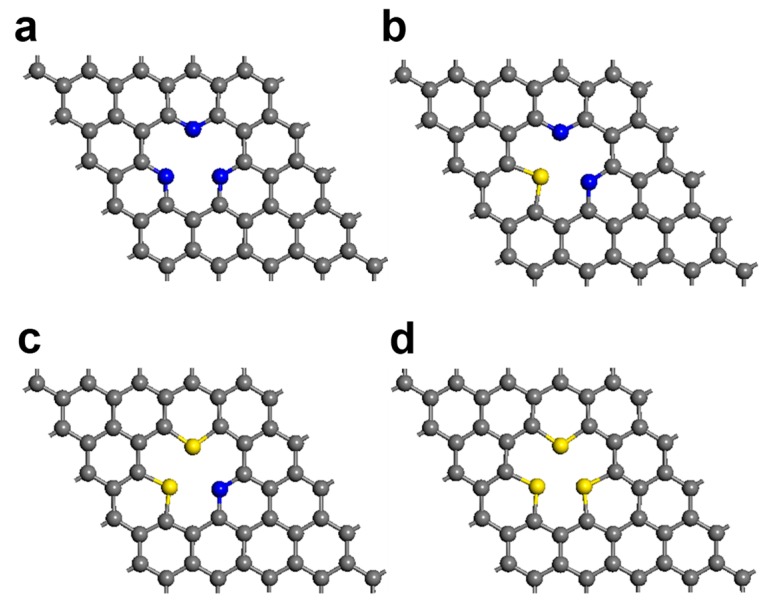
Optimized structures of the (**a**) 3N-gra, (**b**) 2N1S-gra, (**c**) 1N2S-gra, and (**d**) 3S-gra systems. Blue, gray, and yellow denote nitrogen, carbon, and sulfur, respectively.

**Figure 2:**
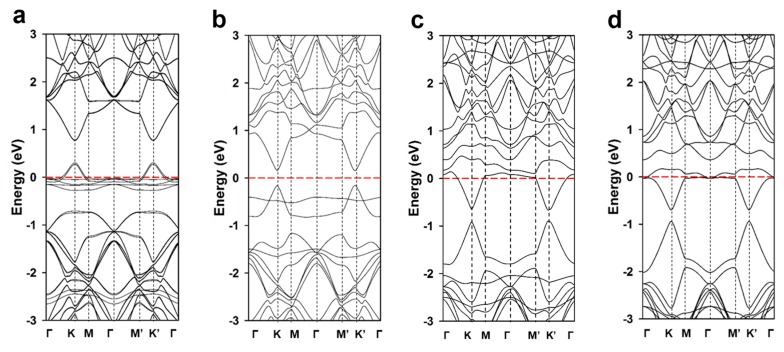
Calculated band structures of the (**a**) 3N-gra, (**b**) 2N1S-gra, (**c**) 1N2S-gra, and (**d**) 3S-gra systems.

Various configurations exist for the doped and defective graphene chosen as the anode material in a lithium-ion battery (LIB). For instance, N-doped graphene exists in distinct forms that include graphitic, pyridinic, and pyrrolic N atoms [47]. Among these nitrogen types, pyridinic N-doped graphene is believed to be associated with high electrocatalytic activity and excellent reversible capacity [29,48,49]. Pyridinic Ns are located at the edges of graphene planes, and arise from sp^2^-hybridized N atoms bonded to two neighboring sp^2^-hybridized C atoms. Using this configuration as the starting point, different configurations of N- and S-doped graphene with mono-vacancy defects were built as simulation models. Three C atoms around a mono-vacancy defect were substituted with different numbers of atomic N and/or S dopants. Regarding model structures in this investigation, research groups successfully reported the synthesis of N- and S-doped graphene [22,43,50,51,52,53,54,55,56,57,58,59,60,61,62,63,64]. In order to analyze the effects of the N and S doping levels on electronic properties, we first fixed the doping concentration to three dopant atoms at the mono-vacancy defect. We constructed four configurations with different N and S doping ratios; the graphene doped with three nitrogen atoms is designated as “3N-gra”, that doped with two nitrogens and one sulfur as “2N1S-gra”, while the graphene doped with one nitrogen and two sulfur atoms is “1N2S-gra”, and the three sulfur-doped graphene is “3S-gra”. The optimized structures of the N- and S-doped graphenes are displayed in Figure 1, with the calculated band structures shown in Figure 2, which reveal clear changes in electronic structure following doping with N and S. Pristine graphene is a zero bandgap semiconductor with its Dirac point located at the Fermi energy [38].

The bandgap clearly opens after doping with N or S, and/or the introduction of a mono-vacancy defect, which is ascribable to the effects of the atomic dopant and/or vacancy defect on the π electrons in the hexagonal rings. Mono-vacancy defects lead to shortages of whole charges compared to pristine graphene, which downshift the Fermi energy, indicating that the mono-vacancy defect acts as a hole dopant with missing π electrons. As shown in Figure S1, the band structure for pristine graphene and graphene with a single vacancy were calculated that the band gap of graphene with a single vacancy is opened at the Dirac point and the Fermi level is downshifted compared to pristine graphene.

On the other hand, N and S atoms have one and two additional valence electrons, respectively; hence, doping with N or S results in an upward shift in the Fermi energy. Table 1 reveals that the bandgap energies also change when the band structures are altered by the atomic dopant and/or vacancy defect. In moving from 3N-gra to 3S-gra, the bandgap energy was observed to gradually decrease with increasing levels of the sulfur dopant. The Fermi level is substantially shifted downward from the Dirac point of pristine graphene in 3N-gra. This downward shift indicates that the 3N-doped graphene exhibits p-type behavior and has an affinity for gaining electron density. In addition, flat bands appeared around the Fermi level. Meanwhile, the Fermi level for 3N-doped graphene is somewhat upshifted compared to the mono-vacancy defective graphene because nitrogen has more available electrons than carbon and can replenish some of the electron deficiency. Nevertheless, doping the mono-vacancy defective graphene with three N atoms is unable to completely compensate for the charge deficiency of the mono-vacancy defect. The energy band gradually becomes narrower, that is to say, the Fermi level is upshifted in moving from 3N-gra to 3S-gra, with increasing levels of the sulfur dopant. Indeed, the energy band for 2N1S-gra is slightly narrower than that of the 3N-gra system. In addition, the band structure of the 1NS2-gra system, which is more doped with sulfur than nitrogen, features visible changes in band energies that are shifted below the Fermi level; hence, this system can be considered to exhibit n-type behavior. Interestingly, the p-type to n-type conversion can be induced through control of the N and S doping levels (e.g., by increasing the S-to-N doping ratio). The 3S-gra system also exhibits n-type character, with slightly downward shifted band energies compared to the 1N2S-gra system; however, the level of downward shift induced by moving from 1N2S-gra to S3-gra is very marginal. The degree in the downward shift in band energy tends to decrease with decreasing nitrogen atom concentration.

**Figure 3:**
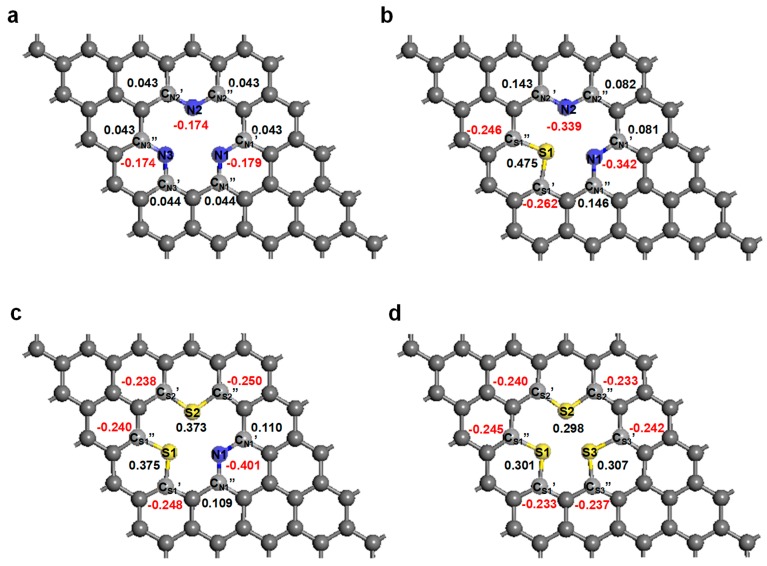
Calculated charge-density distributions on the atoms around the doped mono-vacancy region for the (**a**) 3N-gra, (**b**) 2N1S-gra, (**c**) 1N2S-gra, and (**d**) 3S-gra systems. Blue, gray, and yellow denote nitrogen, carbon, and sulfur, respectively.

Finally, we studied the charge-density distribution of each atom around the mono-vacancy defect for each N- and S-doped graphene system by Mulliken population analysis because the charge distributions on the carbon, nitrogen, and sulfur atoms are important for determining the origin of the alternating electronic properties. Figure 3 displays the charge on each atom around the mono-vacancy defect; positive charges are shown in black, while negative charges are shown in red. The difference in the electronegativity of the N atom (3.04) and the C atom (2.55), which is referred to Pauling scale [65], polarizes the hexagonal ring. Therefore, all of the N atoms inside the mono-vacancy for each system are negatively charged. The average charges on the N atoms in these systems were determined to be −0.176e, −0.341e, and −0.401e for 3N-gra, 2N1S-gra, and 1N2S-gra, respectively. Meanwhile, most of the compensating positive charges are distributed on the adjacent C atoms connected to the atomic N dopants. As shown in Figure 2a (3N-gra system), the charges on the three C atoms connected to the N atoms in the range between +0.043 and +0.044e. Unlike the N-doped systems, the C–S bond is negligibly polarized because the electronegativities of the S (2.58) and C (2.55) atoms are similar. Moreover, the S atom has two additional valence electrons compared to carbon, which provide positive charge and lone pairs of electrons. The average charges on the S atoms in these systems were determined to be +0.475e, +0.374e, and +0.302e for 2N1S-gra, 1N2S-gra, and 3S-gra, respectively. In contrast, most of the compensating negative charges are distributed on the adjacent C atoms bonded to the doping S atoms. As displayed in Figure 2b (the 2N1S-gra system), the C atoms bonded to the N atoms bear positive charges, with values of +0.081e and +0.146e on the C_N1_′ and C_N1_′′ atoms, and +0.143e and +0.082e on the C_N2_′ and C_N2_′′ atoms, respectively. The charges on the C atoms on each side of the N atom are almost identical. On the other hand, the C atoms bonded to the S atom exhibit negative charges, at −0.242e on C_S1_′, and −0.246e on C_S1_′′. Likewise, the 1N2S-gra system showed a similar trend. As shown in Figure 2c, the C atoms bonded to the N atoms, namely C_N1_′ and C_N2_′′, bear charges of +0.109e and +0.110e, which are almost identical. The C atoms adjacent to the S atoms exhibit negative charges, with charges of −0.248e and −0.240e on C_S1_′ and C_S1_′′, and −0.238e and −0.250e on the C_S2_′ and C_S2_′′ atoms, respectively. Finally, the 3S-gra system exhibited charges on the C atoms bonded to the S atoms that were in the −0.245e to −0.233e range. It seems that polarization in the doped region increases with increasing S concentration. The transformed charge-density distribution following doping, as well as the vacancy defect, affects the electronic properties of the graphene system.

## 4. Conclusions

The present density functional theory study aimed to reveal details of the electronic structures of several N- and S-doped graphenes in order to understand the effects of the N and S dopants on the graphene electronic structure. We found that the band structure of graphene can easily be tuned by doping with N and S atoms. The roles of the atomic N and S dopants on the band energies were clearly revealed; these dopants noticeably perturb the band shapes and open the bandgap at the Dirac point, compared to graphene itself. The band energies of 3N-doped graphene were upward shifted below the Fermi level compared with those of pure graphene, and showed p-type behavior. The band structure exhibits a remarkable electronic transition, from “p-type” to “n-type”, in moving from 3N-gra to 3S-gra (with increasing numbers of S atoms) with a downshifting of the band energy below the Fermi level. Moreover, Mulliken population analysis revealed that the atomic N dopants bear negative charges, whereas the atomic S dopants bear positive charges in N- and/or S-co-doped graphene systems, which is ascribable to differences in the electronegativities and numbers of valence electrons among the C, N, and S atoms. In each N- and/or S-co-doped graphene system, all of the N atoms bear negative charges, whereas all of the S atoms bear positive charges. The average charge on the N atoms gradually increases with decreasing numbers of N atoms in the N- and/or S-co-doped graphene system. In contrast, the average charge on the S atoms decreases with increasing numbers of S atoms in the co-doped graphene system. Due to their tunable band-structure properties, the resulting N- and S-co-doped graphenes can be used in energy and electronic-device applications. In conclusion, we expect that doping with N and S will lead to new pathways for tailoring and enhancing the electronic properties of graphene at the atomic level.

## Figures and Tables

**Table 1:** The band gap energies Eg (in eV) for 3N-gra, 2N1S-gra, 1N2S-gra and 3S-gra systems.

	3N-gra	2N1S-gra	1N2S-gra	3S-gra
Bandgap (eV)	0.473	0.350	0.275	0.255

## Supplementary Materials

The following are available online at http://www.mdpi.com/2079-4991/9/2/268/s1, Figure S1: Calculated band structures of the (**a**) pristine graphene and (**b**) graphene with mono-vacancy.
