# Supplementary material for: Tunable Electronic Properties of Nitrogen and Sulfur Doped Graphene: Density Functional Theory Approach
Source: Nanomaterials (Basel). 2019 Feb 15;9(2):268. doi: 10.3390/nano9020268 (PMC6409776; doi:10.3390/nano9020268)
Supplement: Supplementary file 1 [file nanomaterials-09-00268-s001.pdf]

nanomaterials

Supporting Infomation

Tunable Electronic Properties of Nitrogen and Sulfur Doped Graphene: Density Functional Theory Approach

Ji Hye Lee [1], Sung Hyun Kwon [1], Soonchul Kwon [2], Min Cho [3], Kwang Ho Kim [4], Tae Hee Han [5,]* and Seung Geol Lee [1,]*

1. Department of Organic Material Science and Engineering, Pusan National University, 2, Busandaehak-ro 63beon gil, Geumjeong-gu, Busan 46241, Korea; iciti5425@pusan.ac.kr (J.H.L.); rnjstjdgus5@hanmail.net (S.H.K.)
2. Department of Civil and Environmental Engineering, Pusan National University, 2, Busandaehak-ro 63beon gil, Geumjeong-gu, Busan 46241, Korea; sckwon@pusan.ac.kr
3. Division of Biotechnology, Advanced institute of Environment and Bioscience, College of Environmental and Bioresource Sciences, Chonbuk National University, Iksan 54596, Korea; cho317@jbnu.ac.kr
4. School of Materials Science and Engineering, Pusan National University, 2, Busandaehak-ro 63 Beon-gil, Geumjeong-gu, Busan, 46241, Korea; khkim@pusan.ac.kr
5. Department of Organic and Nano Engineering, Hanyang University, Seoul 04763, Korea

* Correspondence: than@hanyang.ac.kr (T.H.H.); seunggeol.lee@pusan.ac.kr (S.G.L.)

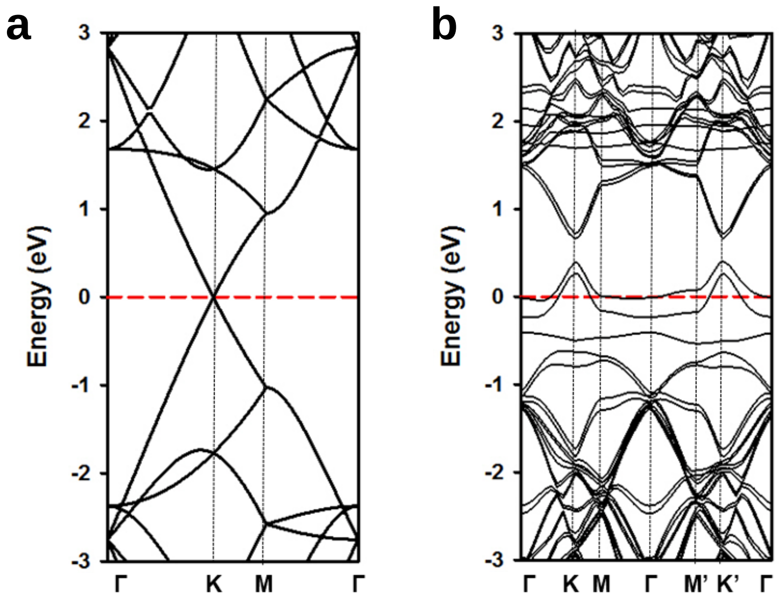

Figure S1. Calculated band structures of the (**a**) pristine graphene and (**b**) graphene with mono-vacancy.
